# Posterior Reversible Encephalopathy Syndrome in an Eclamptic Adolescent: A Case Report

**DOI:** 10.7759/cureus.85287

**Published:** 2025-06-03

**Authors:** Rachel Alef, Amal Akhtar, Maya Barrant, Andrew Carilli, Renee Alexis

**Affiliations:** 1 Osteopathic Medicine, Nova Southeastern University Dr. Kiran C. Patel College of Osteopathic Medicine, Davie, USA; 2 Medicine, Nova Southeastern University Dr. Kiran C. Patel College of Osteopathic Medicine, Fort Lauderdale, USA; 3 Obstetrics and Gynecology, Nova Southeastern University Dr. Kiran C. Patel College of Osteopathic Medicine, Fort Lauderdale, USA

**Keywords:** adolescent pregnancy, eclampsia, high-risk pregnancy, posterior reversible encephalopathy syndrome (pres), problems during pregnancy

## Abstract

Posterior reversible encephalopathy syndrome (PRES) is a neurological condition with diverse etiologies, characterized by transient changes in brain imaging and patient behavior. In this case report, a 13-year-old patient in labor developed eclampsia; hemolysis, elevated liver enzymes, and low platelets (HELLP) syndrome; and ultimately, PRES. PRES is relatively uncommon in adolescents and was likely triggered by the extreme hypertensive stress experienced by this patient. Prompt identification and treatment with antihypertensive and anti-seizure medications led to a relatively rapid resolution of the patient’s postictal, minimally responsive state. Early recognition and management of PRES, particularly in adolescents, are crucial to reducing the risk of long-term complications. Although the long-term outcomes of PRES are not well understood, further research is needed to explore its potential impacts, especially in adolescent populations.

## Introduction

Posterior reversible encephalopathy syndrome (PRES) is a transient neurological condition characterized by a combination of radiologic imaging abnormalities and clinical findings [1]. PRES may also be referred to by other terminologies, such as reversible posterior leukoencephalopathy syndrome, reversible posterior cerebral edema syndrome, posterior leukoencephalopathy syndrome, hyperperfusion encephalopathy, and brain capillary leak syndrome [2]. The condition was first described in 1996, making it relatively novel and likely underdiagnosed [3]. Since its initial characterization, PRES has been reported in individuals across all age groups, though it is more commonly observed in middle-aged women with severe hypertension, renal failure, liver failure, sepsis, certain autoimmune disorders (such as systemic lupus erythematosus), and/or exposure to cytotoxic medications [1,4]. While the pathophysiology is not yet fully understood, the leading theory suggests that uncontrolled hypertension disrupts cerebrovascular autoregulation, resulting in hyperperfusion, endothelial damage, and vasogenic edema [4]. 

Many non-specific clinical features have been identified, all of which may vary in severity, including seizures, headache, visual disturbances, nausea, vomiting, and altered mental status [1,5]. The gold standard for differentiating PRES from other neurologic disorders is brain magnetic resonance imaging (MRI) without contrast, which typically reveals hyper-intense signals, most often in the parieto-occipital lobes, representing vasogenic edema [3]. Prompt identification and treatment of the underlying etiology are essential for the management of PRES [4,6]. With appropriate care, approximately 80% of patients achieve complete recovery within a few days to several weeks [3,4]. However, delayed treatment may result in life-threatening complications such as ischemic injury with associated neurologic deficits, epilepsy, or possible transforaminal cerebellar herniation (reported in children) [1,6]. Although PRES is known to be associated with preeclampsia and eclampsia, its incidence in the general pediatric population is approximately 0.04%, rising to 0.4% among patients in pediatric intensive care units (PICUs) [1,7].

The purpose of this report is to analyze the clinical course of a 13-year-old patient who developed PRES secondary to eclampsia, highlighting the potential underdiagnosis of this syndrome in pediatric populations. The long-term impacts of PRES are not well understood, and prompt management of the underlying pathology, particularly in rare pediatric presentations, may help mitigate these lesser-known long-term effects.

## Case presentation

A 13-year-old female patient, gravida 1, para 0, with no antenatal complications, presented to the emergency department in the middle of the night** **at 33 weeks and 3 days of gestation with complaints of epigastric pain. She was on the verge of a hypertensive emergency, presenting with a blood pressure of 159/104 mmHg. Nifedipine was administered, and she was subsequently admitted. Urine chemistry revealed a creatinine (Cr) level of 27.2 mg/dL and a protein level of 44 mg/dL, with an elevated protein-to-Cr ratio of 1.6. A comprehensive metabolic panel (CMP) showed a low blood urea nitrogen (6 mg/dL)/Cr (0.8 mg/dL) (BUN/Cr) ratio (<20/1) of 6/0.8 (Table 1). Due to epigastric pain, an ultrasound of the liver and gallbladder was performed, which revealed a normal-appearing liver and a gallbladder with a thickened wall. Overnight, the patient's blood pressure remained elevated, and she was scheduled for emergency induction of labor the same day. As the day progressed, although her blood pressure was later stabilized, she developed hemolysis, elevated liver enzyme, and low platelet count (HELLP) syndrome. Laboratory findings included a platelet count of 95,000/mL, aspartate transaminase (AST) of 192 U/L, alanine transaminase (ALT) of 495 U/L, total bilirubin of 2.47 mg/dL, and lactate dehydrogenase (LDH) of 288 U/L. Due to the diagnosis of preeclampsia with severe features, characterized by thrombocytopenia, elevated transaminase levels, and persistent epigastric pain, an emergent cesarean section (C-section) was recommended. Magnesium sulfate was administered preoperatively to prevent seizures, and serum magnesium levels remained within the therapeutic range throughout the perioperative period.

**Table 1:** Pertinent lab values CMP: complete metabolic panel, BUN: blood urea nitrogen, Cr: creatinine, AST: aspartate aminotransferase, ALT: alanine aminotransferase, LDH: lactate dehydrogenase.

Pertinent labs	Patient value	Reference range
Urine creatinine	27.2 mg/dL	0.5-1.0 mg/dL
Urine protein	44 mg/dL	6.0-8.3 mg/dL
Urine protein-to-creatinine ratio	1.6	<0.2
CMP BUN	6 mg/dL	2-20 mg/dL
CMP Cr	0.8 mg/dL	0.5-1 mg/dL
CMP BUN/Cr ratio	6/0.8	10/1-20/1
Platelets	95,000 platelets/mL	150-450,000 platelets/mL
AST	192 U/L	10-40 U/L
ALT	495 U/L	5-55 U/L
Bilirubin	247 mg/dL	<1.5 mg/dL
LDH	288 U/L	100-330 units/L

The patient was given an epidural prior to her delivery. However, immediately before the surgical incision, she experienced a tonic-clonic seizure, despite being on magnesium sulfate. An emergency obstetric code was initiated, and a stat c-section was performed with seizure management provided by anesthesia via IV midazolam. The seizure lasted about 1-2 minutes. The patient had an estimated blood loss of 570 mL, and carboprost tromethamine was administered. The neonate was delivered successfully, but the patient remained in a postictal state and was only responsive to painful stimuli, such as sternal rub. She was not intubated at that time.

**Figure 1:**
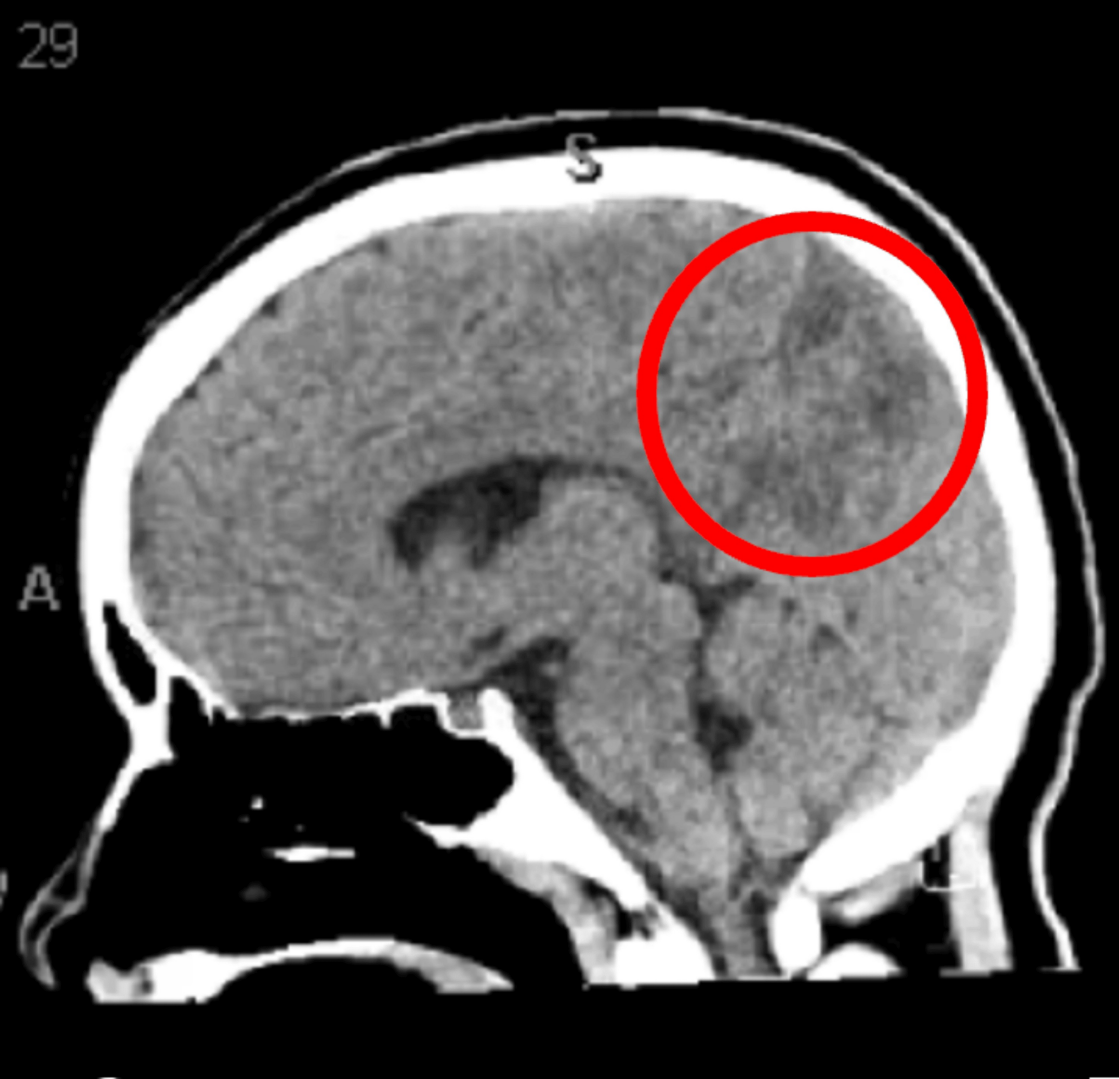
Sagittal non-contrast computed tomography brain scan The red circle represents hypodensity in the right posterior parietal lobe, suggesting posterior reversible encephalopathy syndrome.

Given the patient's minimally responsive condition, she underwent a non-contrast brain computed tomography (CT) scan. During the scan, she experienced a second tonic-clonic seizure, prompting a rapid response. She received lorazepam and was transferred to the pediatric intensive care unit. The CT scan findings were suggestive of PRES (Figure 1), with a hypodense area noted in the right posterior parietal lobe (outlined in red in Figure 1). It was agreed that if the patient experienced a third seizure, endotracheal intubation would be performed.

Magnesium sulfate therapy was continued, with serum levels monitored every three hours to ensure they remained within the therapeutic range. Additionally, levetiracetam was initiated due to the occurrence of seizures despite therapeutic magnesium sulfate. EEG revealed no epileptic activity, and an echocardiogram was unremarkable. The patient also received an RBC transfusion due to blood loss sustained during delivery.

Approximately 10 hours after delivery, the patient was alert but remained mildly confused and not fully oriented. By the time neurology evaluated her, around 14 hours post-delivery, she was reported to be rapidly returning to her neurological baseline. Magnesium sulfate was discontinued that evening, while levetiracetam was continued for an additional day as a precaution. The patient was clinically stabilized from her pregnancy-related complications, had returned to her cognitive baseline, and had a discharge BUN/Cr ratio of 9:0.7. She was discharged in stable condition on postoperative day 4.

## Discussion

PRES incidence

PRES is characterized by several neurological symptoms frequently associated with elevated blood pressure. It is considered a clinical radiographic syndrome that typically develops secondary to an underlying condition [5]. Although numerous cases have been reported, the true incidence of PRES is unknown, especially in the pediatric population [2]. PRES has been documented in individuals ranging from 2 to 90 years old, with a higher prevalence among women [2]. In the general pediatric population, the estimated incidence is 0.04% [8]. Higher rates are observed in children with cancer (0.7%) and those admitted to PICUs (0.4%) [8]. Among pediatric patients with PRES, the reported mortality rate is approximately 3.2% [9]. A 2016 survey of pediatric hospitalizations involving PRES found the mean age of presentation to be 12.5 years, remarkably close to the presenting age of our patient [8]. The most commonly reported etiology of PRES in children, according to a literature review, is sudden elevation in blood pressure [9]. A key limitation in the current body of literature is that most pediatric PRES studies focus on non-pregnant patients, with limited data available on the incidence and risk factors in pregnant adolescents. In contrast, the incidence of PRES in pregnant adults has been more extensively documented.

Several studies have identified eclampsia as a significant risk factor for PRES. One study reported that 97.9% of eclamptic patients who underwent MRI imaging were diagnosed with PRES, while another found the incidence to be 62.5% among eclamptic patients [7,10,11]. Though PRES can also occur in patients with preeclampsia, it appears at a much lower rate compared to patients with eclampsia [11]. In a retrospective study of pregnant women aged 19-37 years, those diagnosed with PRES typically were younger, with a median age of 23 years old, and were primigravid [10]. Notably, adolescents under the age of 15.9 years have been shown to have an increased risk of developing preeclampsia and HELLP syndrome. Given that nulliparity is a recognized risk factor for hypertensive disorders in pregnancy, and considering the increased vulnerability of younger adolescents to these conditions, as well as the mean age of PRES presentation of 12.5 years, our patient, being 13 years old and nulliparous, was likely at an elevated risk for PRES. Nevertheless, PRES remains a rare occurrence in adolescent patients.

Pathophysiology

Similar to its incidence, the exact etiology of PRES remains unknown. Several theories have been proposed, with the most commonly cited mechanisms including autoregulatory failure with resultant hypertension, cerebral ischemia, and endothelial dysfunction. Cerebral autoregulation maintains stable blood flow via arteriolar constriction and dilation. However, in the setting of severe hypertension, this autoregulatory capacity becomes overwhelmed. As arterioles dilate in response to elevated blood pressure, cerebral blood flow increases, potentially leading to hyperperfusion. It is theorized that this hyperperfusion results in the breakdown of the blood-brain barrier, permitting the extravasation of fluid and blood products into the brain parenchyma, thereby causing vasogenic edema [2]. An alternative hypothesis suggests that autoregulatory failure may lead to vasoconstriction, thus leading to focal hypoperfusion, cytotoxic edema, and cerebral infarction. Cerebellar ischemia is thought to be limited to severe cases, as most pathological studies do not demonstrate significant vasoconstriction or ischemia in typical presentations of PRES [2]. The final theory suggests that endothelial dysfunction plays a role in the pathophysiology of PRES, especially in patients with preeclampsia. Studies have shown that biomarkers of endothelial dysfunction, such as elevated lactate dehydrogenase levels and abnormal red blood cell morphology, have a better correlation with the extent of cerebral edema than changes in blood pressure [2]. This theory may be an explanation for the correlation between eclampsia and developing PRES.

Typical presentation

PRES presents with various neurological symptoms, such as headaches, visual impairment, and seizures [5]. PRES typically presents in the setting of acute hypertension, with blood pressures ranging from 160 to 190 mmHg, although 15%-20% of patients with PRES may exhibit normal or slightly elevated blood pressures [1,8]. In children specifically, PRES can occur at even lower pressures, with an average of approximately 140/85 mmHg [8]. Generally, in pediatric cases, tonic-clonic seizures, as observed in our patient, are the most common symptoms and may be preceded by visual impairment. Seizures are more prevalent in pediatric PRES cases compared to adult PRES cases and have an earlier onset. In these cases, bilateral occipital sharp waves are present on EEG during status epilepticus [8]. In more than half of pediatric PRES cases, altered consciousness has been found [8]. Frequently, the occipital lobes are involved in PRES, and patients often have visual disturbances or visual hallucinations [5]. Patients may also present with stupor, somnolence, or coma; these signs of encephalopathy are more prevalent in adult PRES compared to pediatric PRES [5,8]. Because PRES presents similarly to many other neurologic disease processes, such as intracranial hemorrhage, vasculitis of the central nervous system, uremic encephalopathy, and eclampsia, imaging needs to be done to rule out these other processes [1].

Diagnosis

Currently, PRES is a diagnosis of exclusion, but diagnostic criteria have been suggested by Fugate et al. [3]. The suggested diagnostic criteria are acute onset of neurological symptoms, (focal) vasogenic edema, and reversibility of clinical and/or radiological findings [3]. In terms of imaging modality, MRI without contrast is the more sensitive modality; however, CT tends to be used more often in practice [5,12]. On both MRI and CT, vasogenic edema can be seen distributed in a symmetric pattern in the parieto-occipital lobes [5,12]. In reference to Figure 1, the hypodensity in our patient’s right parietal lobe was diagnostic of PRES. EEG, although not used for diagnosis of PRES, may be ordered to detect the presence of epileptic seizures and status epilepticus, like in the case of our patient [5]. There are no serum tests that are specific for PRES [5]. 

Management

In order to manage PRES, it is imperative to identify, treat, and manage the underlying cause; an example would be carefully treating the underlying hypertension. Although no specific antihypertensive regimen is recommended for treating acute hypertension in non-pregnant patients with PRES, treatment is advised when blood pressure surpasses 160/110 mmHg. The target range is typically between 130-150 mmHg systolic and 80-100 mmHg diastolic [1]. Hypertensive patients with PRES may need to be admitted to the intensive care unit (ICU) for careful management of acute hypertension, especially if they have other risk factors or comorbid conditions, such as pregnancy, as seen in this patient [4]. In pregnant patients with eclampsia, treatment should include delivery of the child and magnesium sulfate for seizure prophylaxis [13].

Patients with PRES can sometimes develop critical complications, such as status epilepticus or coma, treatment and management of which necessitate admission to an ICU. There are not many studies identifying a specific anti-seizure regimen for the treatment of seizures in non-pregnant patients with PRES, and there is currently not a specific antiepileptic treatment for these patients, but some anti-epileptic treatments are given during the acute phase of seizures and are discontinued once the seizures have resolved [4,12]. In cases where PRES has led to the complication of epilepsy, the use of antiepileptics will be chronic. If PRES is due to an immunosuppressant, it is recommended to reduce the dose or substitute the agent [4,12].

In this 13-year-old pregnant patient with HELLP syndrome and altered mental status, PRES was suspected to be secondary to complications from eclampsia. The patient experienced two seizures during the course of her care. During and after her seizures, she was managed with multiple medications, including magnesium sulfate, lorazepam, and midazolam. Levetiracetam was also administered prophylactically to prevent further seizures. Although levetiracetam was likely of limited additional benefit, since magnesium sulfate is the standard treatment for eclamptic seizures, it was used due to its common role in managing seizures in pediatric patients, particularly myoclonic seizures [12]. 

Long-term adverse effects of PRES

While the early diagnosis and treatment of PRES have a generally favorable prognosis, long-term complications warrant further study, as mechanisms and prevalence are not fully understood. Some patients, following PRES, may require intensive care and may have long-term effects, including seizures, cognitive problems, and other residual neurological deficits [7,14,15]. A retrospective cohort study demonstrated that PRES was associated with an increased risk of subsequent acute care utilization for seizures, as compared to strokes [15]. There are also fatal complications associated with PRES, including intracranial hemorrhage, ischemic stroke, cerebellar herniation, refractory status epilepticus, cerebral lesions, and brain atrophy [7,14,15]. Some studies have shown a link between vasospasm and ischemia, which could result in these complications. Altered cerebral autoregulation and cerebral vessel rupture have also been linked to the development of cerebral hemorrhage. Damage to the blood-brain barrier, as well as prolonged cytotoxic edema resulting in subsequent cell death, may cause ischemia and hemorrhage [14]. Further research is needed to explore the potential long-term effects of PRES in pediatric patients. 

The recurrence rate of PRES is higher in pediatric patients than in adult patients [16]. PRES in the pediatric population is most commonly due to repetitive hypertension in the setting of chronic renal disease. The long-term risk of unprovoked seizures and epilepsy is rare, unless associated with predisposing premorbid conditions, delayed diagnosis, or improper management. When PRES is recognized and treated quickly, the symptoms and imaging features usually resolve within days to weeks. The treatment of PRES is supportive and consists of rapid reversal of the inciting cause and supportive management of associated complications such as seizures [7]. By promptly recognizing abnormal imaging findings and correlating them with a patient's clinical symptoms, PRES can be diagnosed and treated early, potentially preventing long-term complications such as permanent cerebral lesions and brain atrophy [7]. Seizures were found to be significantly more frequent in the pediatric age group, with an increased incidence in younger children. Visual disturbances were found to be less significant in the pediatric age group than in adults [16]. Furthermore, the lack of studies exploring the factors associated with the development of PRES in pediatric patients specifically leaves room for further studies in these populations as well as the severity of long-term impacts of PRES [16].

## Conclusions

PRES may be an underdiagnosed phenomenon in eclamptic patients, especially within the adolescent population. It is therefore important for clinicians to recognize symptoms associated with PRES to identify and manage the underlying pathology accordingly. Prompt treatment of PRES significantly reduces the risk of long-term complications. Further research is needed to better understand these potential adverse effects, particularly in adolescent patients, as they remain poorly characterized.
